# Novel Docosahexaenoic Acid Ester of Phloridzin Inhibits Proliferation and Triggers Apoptosis in an In Vitro Model of Skin Cancer

**DOI:** 10.3390/antiox7120188

**Published:** 2018-12-11

**Authors:** Theodora Mantso, Dimitrios T. Trafalis, Sotiris Botaitis, Rodrigo Franco, Aglaia Pappa, H. P. Vasantha Rupasinghe, Mihalis I. Panayiotidis

**Affiliations:** 1 Department of Applied Sciences, Northumbria University, Newcastle Upon Tyne NE1 8ST, UK; theodora.mantso@northumbria.ac.uk; 2 Laboratory of Pharmacology, Unit of Clinical Pharmacology, Medical School, National and Kapodistrian University of Athens, 11527 Athens, Greece; dtrafal@med.uoa.gr; 3 Second Department of Surgery, Democritus University of Thrace, 68100 Alexandroupolis, Greece; smpotait@med.duth.gr; 4 Redox Biology Centre, University of Nebraska, Lincoln, NE 68588, USA; rodrigo.franco@unl.edu; 5 Department of Veterinary & Biomedical Sciences, University of Nebraska, Lincoln, NE 68583, USA; 6 Department of Molecular Biology and Genetics, Democritus University of Thrace, 6810Alexandroupolis, Greece; apappa@mbg.duth.gr; 7 Department of Plant, Food, and Environmental Sciences, Faculty of Agriculture, Dalhousie University, Halifax, NS B2N 5E3, Canada; vrupasinghe@dal.ca; 8 Department of Pathology, Faculty of Medicine, Dalhousie University, Halifax, NS B3H 4R2, Canada

**Keywords:** phloridzin, docosahexaenoic acid, flavonoids, skin cancer, melanoma, epidermoid carcinoma, apoptosis, necrosis, cell cycle, oxidative stress, reactive oxygen species

## Abstract

Skin cancer is among the most common cancer types accompanied by rapidly increasing incidence rates, thus making the development of more efficient therapeutic approaches a necessity. Recent studies have revealed the potential role of decosahexaenoic acid ester of phloridzin (PZDHA) in suppressing proliferation of liver, breast, and blood cancer cell lines. In the present study, we investigated the cytotoxic potential of PZDHA in an in vitro model of skin cancer consisting of melanoma (A375), epidermoid carcinoma (A431), and non-tumorigenic (HaCaT) cell lines. Decosahexaenoic acid ester of phloridzin led to increased cytotoxicity in all cell lines as revealed by cell viability assays. However, growth inhibition and induction of both apoptosis and necrosis was more evident in melanoma (A375) and epidermoid carcinoma (A431) cells, whereas non-tumorigenic keratinocytes (HaCaT) appeared to be more resistant as detected by flow cytometry. More specifically, PZDHA-induced cell cycle growth arrest at the G2/M phase in A375 and A431 cells in contrast to HaCaT cells, which were growth arrested at the G0/G1 phase. Elevated intracellular generation of reactive oxygen species ROS was detected in all cell lines. Overall, our findings support the potential of PZDHA as a novel therapeutic means against human skin cancer.

## 1. Introduction

Skin cancer constitutes one of the most common types of cancer in the Caucasian population, with its incidence rates continuously increasing worldwide [1,2,3,4,5]. It is categorized into melanoma (MM) and non-melanoma skin carcinomas (NMSCs), with MM constituting one of the most aggressive and lethal solid tumor types. A large number of reports have associated exposure to UV irradiation with an increased frequency of mutations responsible for MM’s occurrence [6,7]. On the other hand, NMSC involves basal (BCC) and squamous (SCC) cell carcinomas, both of which are different types of keratinocyte carcinomas and are the most common type of malignancies in Caucasians. Incidence of NMSC has also been linked with increased Ultraviolet Radiation (UVR) exposure; however, its metastatic potential and mortality rates remain relatively low compared to MM [1,2,8].

Flavonoids represent a major group of polyphenols whose beneficial effects have been the subject of numerous studies exhibiting their anti-microbial, anti-oxidant, anti-inflammatory, anti-cancer, and anti-mutagenic properties [9,10]. One of the major flavonoids found in apples is phloridzin (phlorizin or phloretin 2′-*O*-glucoside) which, in addition to several other hydrochalcone compounds, has been demonstrated to possess anti-cancer properties against lung, liver, and colon cancer cell lines [11,12,13]. In addition, cryptocaryone as well as other dihydrochalcone compounds were shown to trigger tumor necrosis factor alpha-related apoptosis-inducing ligand (TRAIL)-induced apoptosis in human prostate cancer cells [14,15]. The anti-proliferative effects of chalcone derivatives together with their ability to inhibit their invasiveness have been also documented in breast cancer cells [16]. A recent report described the acylation of flavonoid glucosides (with long chain fatty acids) and their esterification by Lipase B to result in the synthesis of fatty acid esters of phloridzin capable of inducing cytotoxicity in liver, breast, and blood cancer cells together with induction of cell cycle arrest, suppression of DNA topoisomerase IIa, and activation of apoptosis [17,18]. Interestingly, decosahexaenoic acid ester of phloridzin (PZDHA) was found to be the most promising, amongst the other ester compounds, thus its effects were further explored in subsequent studies. More specifically, PZDHA was shown to possess anti-inflammatory properties in THP-1 differentiated macrophages following exposure to lipopolysaccharide (LPS) [19]. Moreover, it was demonstrated that PZDHA selectively induced the killing of breast cancer cell lines whereas non-malignant breast cells remained unaffected. Additional experiments with breast cancer cell lines and breast cancer xenograft models indicated induction of cell cycle growth arrest (at G2/M phase), activation of apoptosis, and overall suppression of tumor growth [20].

However, the effects of PZDHA on experimental models of skin cancer have not yet been investigated. To this end, our in vitro model of skin cancer consisted of two different skin cancer types, namely A375 (MM) and A431 (NMSC) cell lines as well as a non-tumorigenic immortalized keratinocyte (HaCaT) cell line. The latter was used in the context of providing a control, a non-malignant cell type which predominantly exists in the epidermis in addition to the basal layer of the skin (basal keratinocytes) [21]. Our hypothesis is that the cancer skin cell lines would be selectively more sensitive to cytotoxicity-induced by PZDHA when compared to the non-tumorigenic cell line. We would expect such observations to be recorded as lower concentration of the compound that gives half-maximal response (EC50) values and/or higher levels of other cytotoxicity endpoints such as those of apoptosis, necrosis, oxidative stress, and cell cycle growth arrest. In support of our hypothesis, previous studies have demonstrated that exposure to PZDHA is cytotoxic in liver and breast cancer cells while non-malignant hepatocytes and mammary cells remained unaffected [18,20]. Therefore, in the present study we aimed to examine the potential role of PZDHA in an in vitro model of skin cancer by assessing its potential to act as a selective cytotoxic agent in inducing cell cycle growth arrest, generation of oxidative stress, and activation of cancer cell death. Such approaches are justified in the context of developing targeted therapeutic approaches aiming to utilize compounds/agents with the capacity to selectively induce cytotoxicity in cancer cells while non-tumorigenic cells are either not affected or more resistant.

## 2. Materials and Methods

### 2.1. Chemicals, Reagents, and Test Compound

Dulbecco’s Modified Eagle’s Medium (DMEM), phosphate buffer saline (PBS), fetal bovine serum (FBS), trypsin, penicillin/streptomycin, and l-glutamine were obtained from Labtech International Ltd. (Sussex, UK). The CellEvent Caspase 3/7 Green flow cytometry assay kit, FxCycle PI/RNase staining solution, and DAPI dye were supplied by Thermo Scientific (Waltham, MA, USA). Dihydrorhodamine 123 (DHR 123) and resazurin sodium salt were purchased from Sigma–Aldrich (St. Louis, MO, USA). All chemicals were of analytical grade and obtained from Invitrogen (Carlsbad, CA, USA), Applichem (Darmstadt, Germany), and Sigma–Aldrich. Synthesis of PZDHA based according to Ziaullah et al. [17]. Stock solutions (100 mM) were prepared in DMSO and stored at −20 °C.

### 2.2. Cell Lines

The human malignant melanoma (A375) and epidermoid carcinoma (A431) cell lines were purchased from Sigma–Aldrich (St. Louis, MO, USA). The human immortalized keratinocyte (HaCaT) cell line was kindly provided by Dr. Broby (Dermal Toxicology and Effects Group; Public Health England, UK). All cell lines were maintained in DMEM medium with high glucose content, supplemented with 10% FBS, 2 mM l-glutamine, and 1% pen/strep (100 U/mL penicillin, 100 μg/mL streptomycin). Cells were cultured in a humidified atmosphere at 37 °C and 5% CO_2_, grown as monolayers and sub-cultured at 80–90% confluency. All cell lines were cultured for 15–20 passages before new stocks were utilized. Cell culture media and reagents were purchased from Labtech (East Sussex, UK) whereas all cell culture plasticware were obtained from Corning (Corning, NY, USA).

### 2.3. Cell Viability Assay

The Alamar-blue assay was utilized where resazurin was converted to resorufin in metabolically active cells with the resulting elevation in absorbance being proportional to the levels of viable cells. Briefly, A375, A431, and HaCaT cells were seeded into 96-well plates in 100 µL/well and incubated overnight prior to exposure to PZDHA. Cell density of A375 was 8000 and 4000 cells/well and for A431 and HaCaT 10,000 and 5000 cells/well for 24 and 48 h, respectively. On the following day, cells were exposed to a range of concentrations (1, 10, 25, 50, 75, and 100 µM) over different incubation periods. For control conditions, cells were incubated with complete medium only or medium containing 0.1% DMSO (vehicle). At the indicated time points, fresh medium (containing 0.1 mg/mL resazurin) was added into each well and incubated for 2–4 h (depending on the type of cancer cell line), at 37 °C. The plates were then centrifuged, and absorbance was recorded at 570 nm and 600 nm (reference wavelength) using a Spark multimode plate reader (Tecan, Switzerland). The levels of cell viability were estimated and expressed as percentage of control cells.

### 2.4. Determination of Apoptosis by Flow Cytometry

The CellEvent Caspase 3/7 Green flow cytometry assay kit was utilized for the detection of apoptosis according to the manufacturer’s instructions. Briefly, cells were plated into 100 mm dishes and allowed to adhere overnight. Cell density of A375 was 1.4 × 10^6^ and 0.7 × 10^6^ cells per dish and for A431 and HaCaT 1.5 × 10^6^ and 0.8 × 10^6^ cells per dish for 24 and 48 h, respectively. The next day, cells were exposed to PZDHA for 24 or 48 h. Next, cells were harvested, washed twice with PBS and a single cell suspension of 10^6^ cells/mL was prepared. Then, 0.5 µL of CellEvent Caspase 3/7 Green detection reagent was added into 0.5 mL of each cell suspension and samples were incubated at 37 °C for 30 min. Five min prior to the end of the incubation period, 1 µM of DAPI was added. Data acquisition and analysis of 20,000 events, for each sample, was performed using a FACS Canto II (BD Biosciences, San Jose, CA, USA) flow cytometer. Caspase-3/7-positive cells were identified as apoptotic and DAPI-positive cells as necrotic.

### 2.5. Morphological Observation under Inverted Phase Contrast Microscope

A375, A431, and HaCaT cells were seeded in 100 mm dishes and exposed to either vehicle (0.1% DMSO) or 70 µM PZDHA for 24 and 48 h. The density of A375 cells was 1.4 × 10^6^ and 0.7 × 10^6^ per dish while for A431 and HaCaT cells was 1.5 × 10^6^ and 0.8 × 10^6^ per dish for 24 and 48 h, respectively. At the indicated time points, the morphology of cells was observed by an inverted phase contrast microscope (ZOE fluorescent cell imager, Bio-rad, Hercules, CA, USA) and images were captured at 20× magnification.

### 2.6. Cell Cycle Analysis by Flow Cytometry

The FxCycle PI/RNase staining solution was used according to the manufacturer’s instructions. Following exposure to PZDHA, cells were harvested and washed twice with PBS. The density of A375 cells was 1.4 × 10^6^ and 0.7 × 10^6^ per dish while for A431 and HaCaT cells was 1.5 × 10^6^ and 0.8 × 10^6^ per dish for 24 and 48 h, respectively. Approximately 0.5 × 10^6^ cells were fixed in cold 70% ethanol, for 1 h or longer, at 4 °C until further processed. Cells were then washed twice with PBS to remove ethanol and finally suspended in FxCycle PI/RNase staining solution for 30 min at room temperature (RT) in the dark. Data acquisition and analysis of 10,000 events, for each sample, was performed using a FACS Canto II (BD Biosciences) flow cytometer.

### 2.7. Measurement of Intracellular ROS

After cells were exposed to PZDHA, they were harvested and washed twice with PBS. The density of A375 cells was 1.4 × 10^6^ and 0.7 × 10^6^ per dish while for A431 and HaCaT cells was 1.5 × 10^6^ and 0.8 × 10^6^ per dish for 24 and 48 h, respectively. A single cell suspension of 10^6^ cells/mL was prepared and dihydrorhodamine 123 (DHR 123; 10 µM) was added and incubated for 5 min at 37 °C. One (1) µM of DAPI was also added to each sample and incubated for 5 min for staining of dead cells. Data acquisition and analysis of 10,000 events, for each sample, was performed using a FACS Canto II (BD Biosciences) flow cytometer. Finally, DAPI positive cells were excluded from further analysis of the results.

### 2.8. Statistical Analysis

All data were expressed as mean values ± SD and comparisons were made between control and PZDHA-exposed groups. Statistical analyses were performed by one-way ANOVA with Tukey’s test for multiple comparisons after using the SPSS v.22 software. Statistical significance was set at *p* < 0.05. Finally, EC50 values were calculated utilizing the Sigma Plot v12.5 software.

## 3. Results

**Figure 1:**
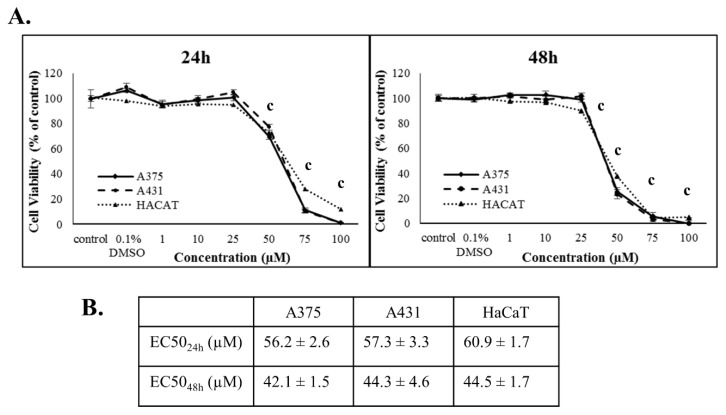
Cytotoxicity of PZDHA in an in vitro model of skin cancer. Viability curves (**A**) and EC50 values (**B**) after exposure to PZDHA. Briefly, A375, A431, and HaCaT cells were exposed to various concentrations of PZDHA (1, 10, 25, 50, 75, and 100 µM) for 24 and 48 h. Cell viability was determined by utilizing the Alamar-blue assay. Data are expressed as percentage of control cells and are presented as means ± SD (*n* = 5). Data are representative of two independent experiments. Finally, (c) represents statistical significance set at *p* < 0.001.

Cytotoxicity of PZDHA was investigated in an in vitro model of skin cancer consisting of human malignant melanoma (A375), epidermoid carcinoma (A431), and immortalized non-tumorigenic keratinocyte (HaCaT) cell lines. Initial experiments involved the determination of viability curves in all three cell lines following exposure to various concentrations of PZDHA over different incubation periods. According to our results, PZDHA induced cytotoxicity in a dose- and time-dependent manner in all three cell lines and to a similar extent (Figure 1A). Concentration of the compound that gives half-maximal response EC50 values were determined to be 56.2, 57.3, and 60.9 µM, while they were reduced to 42.1, 44.3, and 44.5 µM after 24 h and 48 h of incubation with PZDHA in A375, A431, and HaCaT cells, respectively (Figure 1B).

**Figure 2:**
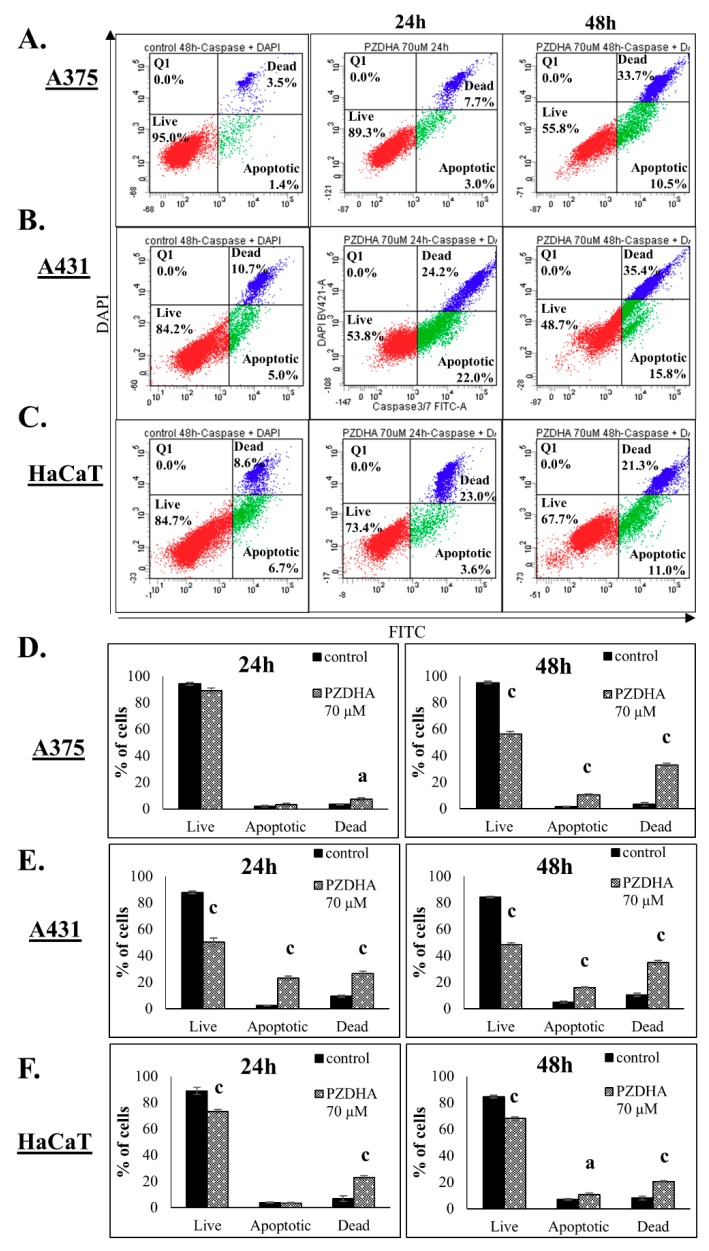
The effect of PZDHA on apoptotic induction in an in vitro model of skin cancer. Dot-blots of A375 (**A**), A431 (**B**), and HaCaT (**C**) cells assessed for caspase 3/7 activation. Cells were treated with 70 µM PZDHA for 24 and 48 h and subsequently incubated with DEVD-substrate and DAPI for the detection of apoptotic and dead cells respectively. Quantification of live, apoptotic, and dead subpopulations in A375 (**D**), A431 (**E**), and HaCaT (**F**) cells treated with 70 µM PZDHA for 24 and 48 h. Data are presented as means ± SD (*n* = 3) and are representative of two independent experiments. Finally, (a) represents statistical significance set at *p* < 0.05, and (c) at *p* < 0.001.

Next, we determined the activation of cell death in response to PZDHA exposure at concentrations near to EC50 values in A375 cells. In doing so, there was no significant activation of apoptosis (monitored as active caspase 3/7 levels) nor necrosis (determined as DAPI-positive staining) at 50 µM PZDHA. Exposure at 70 µM PZDHA resulted in non-significant changes in the population of dead cells, at 24 h, but at 48 h there was a remarkable decline in the rates of live cells accompanied with increased apoptotic and necrotic levels, respectively (Figure 2A,D). In comparison, A431 cells were more sensitive as there was a profound decrease in cell viability levels while both apoptosis and necrosis increased respectively, at 24 h of exposure, (Figure 2B,E) followed by an even more profound effect after 48 h of exposure (Figure 2B,E). Interestingly, HaCaT cells were found to be more resistant compared to both cancer cell lines, throughout the entire exposure period (Figure 2C,F). At the same time, there was an increase of apoptosis and necrosis at both time courses (Figure 2C,F). Overall, it was apparent that PZDHA triggered cell death cascades, in all three cell lines, with A431 cells being more sensitive and HaCaT more resistant when compared to A375 cells.

**Figure 3:**
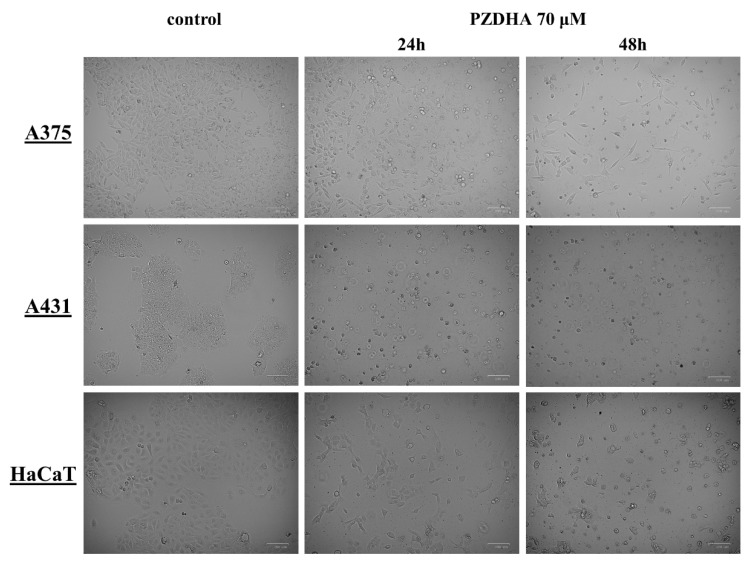
Morphological changes of cells upon exposure to PZDHA. Representative images of A375, A431, and HaCaT cells treated either with vehicle (0.1% DMSO) or PZDHA (70 µM) for 24 and 48 h. Images were recorded by utilizing the ZOE fluorescent cell imager at 20× magnification.

In addition, we observed morphological alterations in all cell lines following exposure to 70 µM PZDHA for 24 and 48 h by utilizing inverted phase contrast microscopy. Such exposure to PZDHA had a significant effect on the confluency levels of all cell lines, however HaCaT appeared to be less affected compared to A375 and A431 cells (Figure 3). Also, there appeared to be PZDHA-induced alterations on the shape and morphology of all cell lines as cellular membrane structure appeared to be distorted causing cells to shrink and consequently detach from the plates.

**Figure 4:**
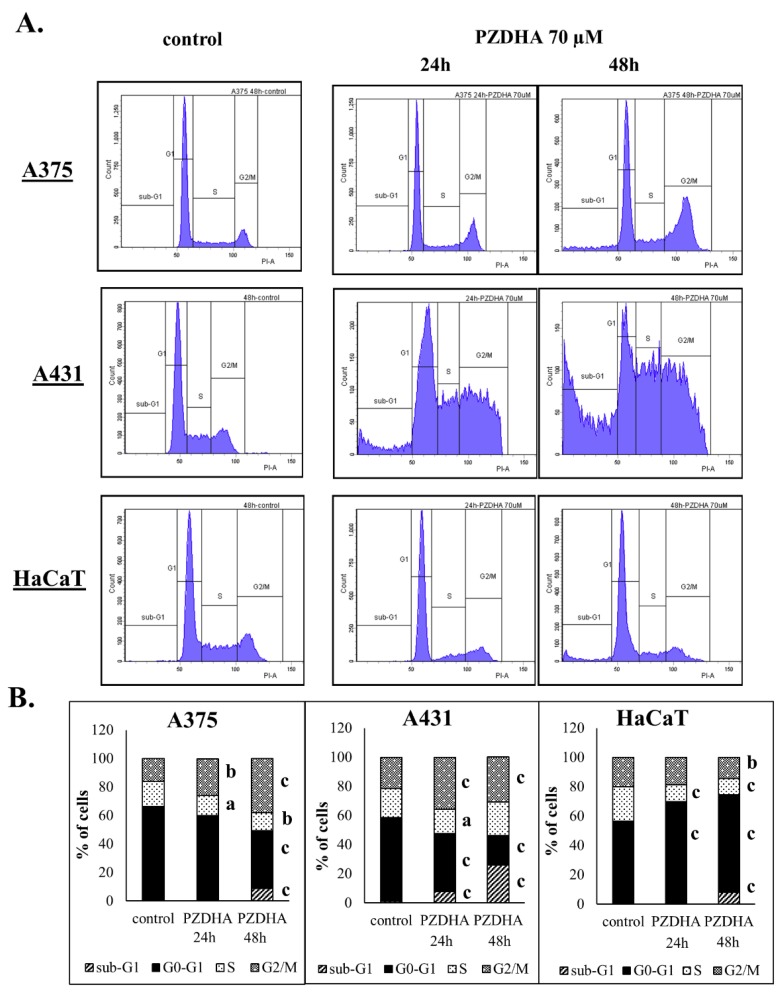
The effect of PZDHA on cell cycle distribution in an in vitro model of skin cancer. Histograms show cell cycle distribution of A375, A431, and HaCaT cells in response to 70 µM PZDHA exposure for 24 and 48 h (**A**). Quantification of cell subpopulations under different cell cycle phases (e.g., sub-G1, G0/G1, S, G2/M) (**B**). Data are presented as means ± SD (*n* = 3) and are representative of two independent experiments. Finally, (a) represents statistical significance set at *p* < 0.05, (b) at *p* < 0.01, and (c) at *p* < 0.001.

Moreover, exposure of A375 cells to PZDHA, over 24 h, led to very minor changes in the contents of G2/M and S phases, while at 48 h there were more substantial changes occurred including a major shift to the G2/M phase (Figure 4A,B). Similarly, PZDHA induced G2/M and S phases growth arrest in A431 cells accompanied by a marked increase in sub-G1 population throughout the entire time course (Figure 4A,B). Surprisingly, HaCaT cells exhibited a growth arrest in G0/G1 phase under both exposure periods (Figure 4A,B). Overall, our data suggest a different pattern of cell cycle distribution between tumorigenic (A375 and A431) and non-tumorigenic (HaCaT) cells.

**Figure 5:**
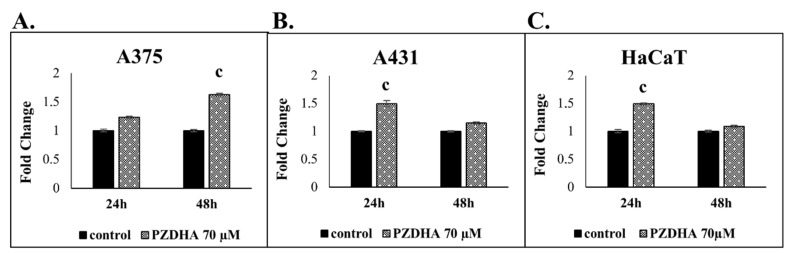
The effect of PZDHA on generation of oxidative stress in an in vitro model of skin cancer. Quantification of ROS generation in response to 70 µM PZDHA over 24 and 48 h of exposure in A375 (**A**), A431) (**B**), and HaCaT (**C**) cells. Data are expressed as fold change compared to control cells and presented as means ± SD (*n* = 3). Data are representative of two independent experiments. Finally, (c) represents statistical significance set at *p* < 0.001.

Finally, we evaluated generation of ROS as a response to PZDHA exposure and our data demonstrated that there was induction of oxidative stress in all three cell lines. However, ROS generation in A375 cells was more profound (at 48 h) as indicated by a 1.7-fold change, whereas in A431 and HaCaT cells it was found to be equal to 1.5-fold at 24 h and dropping to control levels at 48 h (Figure 5A–C).

## 4. Discussion

According to our results, PZDHA was shown to be cytotoxic in all three cell lines. However, HaCaT cells were shown to be more resistant, at higher concentrations of the compound, compared to both cancer cell lines (Figure 1A). In addition, EC50 values were equal to approximately 60 and 40 µM for 24 and 48 h, respectively in all cell lines (Figure 1B). These observations are in agreement with the current literature as this derivative of phloridzin has been previously shown to exert cytotoxicity against liver and breast carcinomas while not affecting non-malignant cell lines of the same tissue type. In addition, the observed EC50 values followed the same range of concentrations as in our experimental model [18,20]. In order to investigate further into the anti-proliferative effects of PZDHA, in our model, we looked into the mode of cell death activation. Our findings confirmed triggering of both apoptosis and necrosis, in all three cell lines, following treatment with 70 µM PZDHA but to a different extent depending on the cell type (Figure 2A–F). More specifically, A431 cells exhibited higher levels of apoptosis and necrosis compared to A375 cells. In comparison, HaCaT cells were more resistant amongst all three cell lines (Figure 2A–F). The participation of the apoptotic cascade via caspase-3/7 activation has been also shown in other in vitro cancer models, including breast, liver, and blood, in response to PZDHA treatment [18,20,22]. Numerous other reports have documented the ability of a wide range of flavonoids to stimulate apoptosis in various cancer types, thus indicating their chemo-preventive properties [23,24]. On another note, it is worth mentioning that PZDHA has been shown to inhibit tyrosinase compared to its parental compound (phloridzin) [17]. To this end, melanocytes are capable of producing melanin (responsible for protecting cells by absorbing UV radiation) while l-tyrosine positively regulates melanogenesis and augments melanocytes’ metastatic potential [25,26]. Elevated levels of melanin, in metastatic melanoma cells, have been linked to the progression of the disease while also having a negative impact on the effectiveness of radiation therapy [27]. Several flavonoids have been studied for their ability to act either as positive or negative regulators in melanogenesis; however, the exact mechanisms of how they exert their anti-proliferative and anti-metastatic properties are not fully elucidated [28]. Nevertheless, there is encouraging evidence on the beneficial effects of several flavonoids by their conjunction with nanoparticles for their targeted delivery in in vitro and in vivo models of melanoma [29,30,31,32]. Hence, PZDHA could potentially act as a new and promising approach in melanoma treatment. Moreover, when assessing cell cycle distribution after exposure to PZDHA a quite distinct pattern was evident characterized by a G2/M growth arrest phase in A375 and A431 cells in contrast to a G0/G1 growth arrest phase in HaCaT cells (Figure 4A,B). Evidence from a number of other studies has indicated that different flavonoids are associated with a G2/M growth arrest phase in various cancer cell lines [33,34]. However, such effect depends on the action of the specific flavonoid and the particular cancer cell type used in each case since other studies have shown the existence of a G0/G1 growth arrest phase as well [35,36]. In particular, exposure of hepatoma (HepG2) cells to PZDHA was shown to induce growth arrest in G0/G1 phase [18], whereas PZDHA exposure to breast carcinoma (MDA-MB-231) cells caused growth arrest in G2/M phase [20]. In addition, the fact that both A375 and A431 cells have a wild-type p53 status [37,38] while HaCaT cells have a p53-mutated one [39] suggests that such observed differences in cell cycle distribution could, perhaps, be attributed to p53 status. Finally, although PZDHA-induced stimulation of oxidative stress was evident in all three cell lines, there was a differential response in ROS production based on the cell type itself and in the context of being either a melanoma (A375) or a non-melanoma (A431) or a keratinocyte (HaCaT) one (Figure 5A–C). In addition, induction of oxidative stress can be linked to alterations in cell cycle distribution [40] as well as activation of cell death [41]. Moreover, although the antioxidant properties of PZDHA have been previously documented [17], it is widely known that high concentrations of antioxidants can result in the induction of oxidative stress as a consequence of pro-oxidant effects [42].

## 5. Conclusions

Collectively, in the present study we have provided evidence that a novel polyphenol fatty acid ester derivative, PZDHA, is cytotoxic against melanoma (A375) and non-melanoma skin cancer (A431) cells, while non-tumorigenic keratinocyte (HaCaT) cells appeared to be more resistant. Furthermore, we demonstrated the occurrence of such a distinct cytotoxicity profile in the context of activated apoptosis and necrosis, induced alterations in cell cycle distribution, as well as increased generation of oxidative stress. In conclusion, our study provides preliminary evidence supporting the potential of PZDHA as a novel therapeutic agent against human skin cancer.
